# Use of the physical environment to support everyday activities for people with dementia: A systematic review

**DOI:** 10.1177/1471301216648670

**Published:** 2016-08-04

**Authors:** R Woodbridge, MP Sullivan, E Harding, S Crutch, KJ Gilhooly, MLM Gilhooly, A McIntyre, L Wilson

**Affiliations:** Brunel University, UK; University College, UK; Brunel University, UK

**Keywords:** dementia, environmental intervention, activities of daily living, quality of life, Alzheimer’s disease, physical environment

## Abstract

Difficulty with everyday activities is a key symptom and defining feature of dementia, relating to subjective reports of well-being and overall quality of life. One way to support individuals in their daily activities is by modifying the physical environment to make it easier to interact with during activity performance. This systematic review explores the range of studies available using physical environmental strategies to support performance in everyday activities for people with dementia. Seventy-two relevant studies were identified by the search. Physical environmental strategies included changes to the global environment and to architectural features, use of moveable environmental aids and tailored individual approaches. Strategies supported general everyday activity functioning (N = 19), as well as specific activities, particularly mealtimes (N = 15) and orientation in space (N = 16); however, few studies were found that focused on aspects of personal care such as dressing (N = 1) and showering or the preferred hobbies of individuals (N = 0). Overall, there appeared to be a lack of research within private home environments, and of studies which specify the dementia syndrome or the whole neuropsychological profile of people with dementia. More work is needed to extend theoretical understandings of how people with dementia interact with their environments so that these spaces can be designed to further support activities of daily living performance. Future work in this field could also incorporate the perspectives and preferences of those living with dementia.

## Background

Dementia is one of the leading health and social economic challenges of our age (World Health Organisation, 2012) with 47.47 million people worldwide estimated to be living with dementia and this number projected to rise to 135.46 million by 2050 (Prince, Guerchet et al., 2013). Whilst there is no cure, understanding how to support people to live well with dementia is of key importance. The ability to engage independently in everyday activities is closely linked to feelings of well-being for people with dementia (Andersen, Wittrup-Jensen, Lolk, Andersen, & Kragh-Sørensen, 2004) and to reduced carer burden for family members (Potkin, 2002). Therefore, one way to support people living with this disorder, and carers, is to understand difficulties with daily activities and develop strategies to support them.

Previous research has shown that functioning in everyday tasks is affected early in dementia and this has been classified as a defining feature of the disorder (DSM-IV criteria). Activities of daily living (ADL) scales have been developed and standardised to clinically assess these difficulties. Scales typically include measures of basic ADLs (BADLs) or self-care tasks such as eating, drinking and dressing, and measures of instrumental ADLs (IADLs) or more complex everyday tasks such as using the telephone, shopping and participating in hobbies. For example, the Bristol Activities of Daily Living Scale (BADLS) (Bucks, Ashworth, Wilcock, & Siegfried, 1996) developed in the UK includes 20 items assessing both BADLs and IADLs, which carers perceive as challenging for people with dementia. Typically, the more complex IADLs have been found to be impaired early in dementia (Barberger-Gateau, Dartigues, & Letenneur, 1993), followed by BADLs as the disease progresses (Mohs, Schmeidler, & Aryan, 2000). However, these findings should be taken with caution as some research has found significant differences in performance for each IADL (Giebel, Challis & Montaldi, 2014) and BADL item (Giebel, Sutcliffe, & Challis, 2015) and variation across individuals with dementia.

People with dementia can be supported in their ability to perform everyday activities, for example, with behavioural therapy (Rogers et al., 1999; Staal et al., 2007), social support (Phinney, 2006) and cognitive training (Zanetti et al., 1997). Predominantly, however, the focus in the literature is on modifying the external physical environment to make it easier to interact with during activity performance. Theoretically, this is often justified using the ‘Press-Competence Model’ (Lawton & Nahemow, 1973). This model suggests that as competence declines, as in dementia, with a progressive deterioration in various functions, the environment places increasing demands on an individual. These demands are thought to cause maladaptive behaviours, or as evidenced above, for people with dementia, increasing difficulties with performing everyday tasks. Applying Powell-Lawton’s theory, modifying the external environment may help to maintain the ‘maximum performance potential’ for individuals, supporting their independence and quality of life in everyday activities for longer.

Existing reviews on physical environmental interventions for people with dementia demonstrate the positive effects some strategies can have on quality of life (Day, Carreon, & Stump, 2000; Fleming, Crookes, & Sum, 2008; Gitlin, Liebman, & Winter, 2003), affect and behaviour (Beck, 1998; Cohen-Mansfield, 2001; Daykin, Byrne, Soteriou, & O’Connor, 2008; Padilla, 2011) and spatial/perceptual abilities (Caffò et al., 2013; Marquardt, 2011; Letts et al., 2011). For example, Padilla (2011) reviewed 33 papers and found features such as a visually complex environment, music and light therapy reduced agitation and negative behaviours. There is some debate around the definition of the ‘physical environment’ used within existing reviews (Gitlin et al., 2003). For the purpose of this paper, we follow the definition of the ‘physical layer’ of the environment provided by Harris, McBride, Ross and Curtis (2002) to include the ambient environment (lighting, noise, temperature), architectural aspects (permanent features, e.g. physical layout) and interior design features (less permanent aspects, e.g. layout, furnishings, objects and design).

Arguably, carrying out everyday tasks may be particularly well supported by environmental strategies given that they involve direct interaction with the external environment. Nevertheless, there is a knowledge gap in terms of understanding the scope and evidence for environmental strategies supporting performance in various everyday activities. Only one previous review was identified on this topic (van Hoof, Kort, van Waarde, & Blom, 2010); however, this article did not address the full range of everyday activities and focused on good practice guidelines rather than evidence-based studies.

The present review is intended to fill the knowledge gap and address the following questions: (1) How is bodily performance in everyday activities supported by evidence-based research using the physical environment? (2) What is the breadth of this research in terms of the activities that are being supported and the dementia ‘populations’ and settings that are included? Given evidence which suggests the majority of daily activities take place within the living dwelling for people with dementia (Evans, 2003), the review is focused on research in the private home/residential environment as opposed to internal or external public spaces.

## Method

The review includes a diverse range of studies with different research designs which called for an integrative and flexible methodology for the evidence to be synthesised and appraised appropriately. Content analysis was applied in the first instance to group and code environmental strategies identified in the articles to support pre-described everyday activity categories. To do this the BADLS (Bucks et al., 1996) with 20 daily activities was used as a framework and each study was assigned a code (1–20) according to the category it fitted. This scale was selected as it covers a range of IADLs and BADLs which are reported to be affected from early in the disease by carers (Bucks et al., 1996). Types of environmental strategies for each activity category are summarised and descriptively appraised in the ‘Findings’ section.

### Search strategy

A comprehensive literature search was conducted from September 2014 to March 2015. This involved searches using the following electronic databases: *Scopus, CINAHL, Google Scholar, Web of Science, PubMed* and *PsycInfo.* A combination of search terms is used including: ‘*dementia*’ OR ‘*alzheimer’s*’ AND ‘*activities of daily living*’ OR ‘*ADLs*’ OR specific ADL terms from BADLS (e.g. ‘*dressing*’, ‘*hobbies*’, ‘*orientation in time*’) AND ‘*physical environment*’ OR ‘*environmental adaptation/changes/modifications*’, ‘*design intervention*’, ‘*assistive technology*’. The following relevant journals were also hand searched: ‘*Social Care Online*’, ‘*Dementia*’, ‘*American Journal of Alzheimer’s Disease and Other Dementias*’, ‘*Environment and Behaviour*’, ‘*Journal of Environmental Psychology*’, ‘*The Gerontologist*’, ‘*Alzheimer’s Care Quarterly*’, ‘*Ageing and Society’.* This was carried out to ensure all relevant articles were picked up by the search terms used. A final manual searching of reference lists from existing literature reviews on environmental strategies and dementia was conducted and appropriate references were added to the catalogue.

### Selection criteria

Articles were included if they met the following inclusion criteria:

1. The population of interest was people with a diagnosis of dementia.
2. The variable of interest was some element of the ‘physical environment’ here defined as everyday design aspects within the living space such as ambient features (e.g. music/lighting), interior design features (e.g. furnishings and less permanent objects) and architectural features (permanent features, e.g. altering the spatial layout) (similar to Harris et al. (2002)). This included assistive technology and devices which affect the physical design of the environment as opposed to items or articles belonging to the person (e.g. wheelchairs or special clothing).
3. The study included at least one outcome measure relating to the ability of the person with dementia to bodily perform one or more everyday activities.
4. The study was conducted in a real-world setting where the person with dementia spends a sufficient amount of time carrying out everyday tasks (i.e. day centre, home, care home).
5. The study was published in English.

### Data synthesis

**Figure 1.**
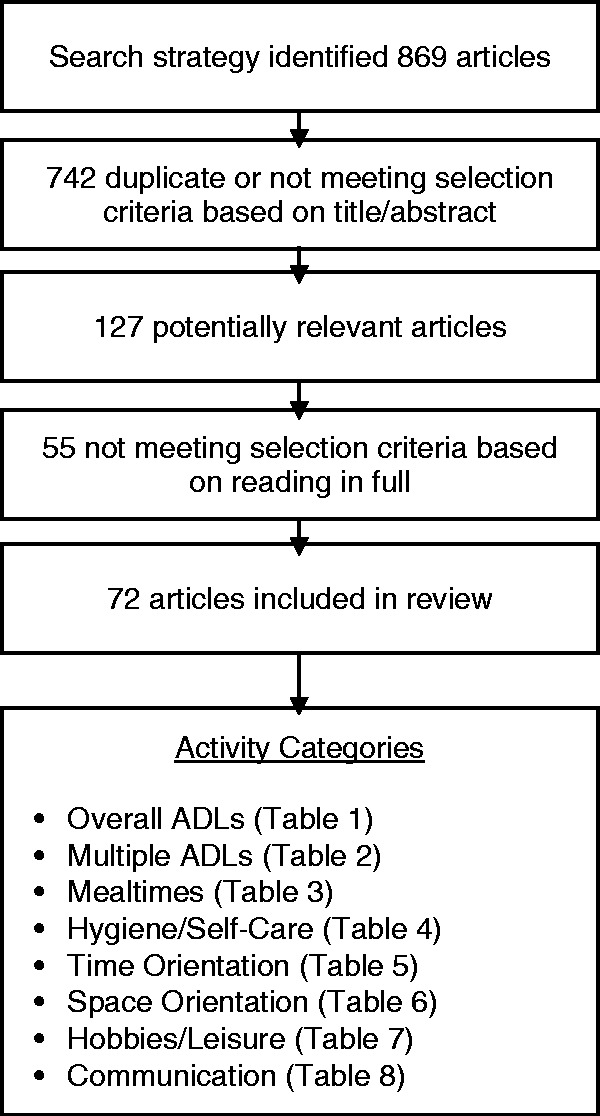
Flow diagram showing results from search strategy and categories of daily activities supported by physical environmental strategies.

An online reference manager (Refworks) was used to keep track and sort references during the literature search period. All studies which met the inclusion criteria were read in full and classified into everyday activity categories (see Figure 1). The articles were then split evenly amongst three authors (RW, MPS, EH) and each study was summarised into a data extraction form (Coren & Fisher, 2006). The team then came together to review the forms and arrive at a consensus on types of environmental strategies and to descriptively appraise the findings.

## Findings

The systematic search identified a total of 869 results, of which 72 papers were considered relevant for final review (Figure 1). The ‘Findings’ section is organised into subheadings of activity categories with themes of environmental interventions for each grouping.

### Study characteristics

**Figure 2.**
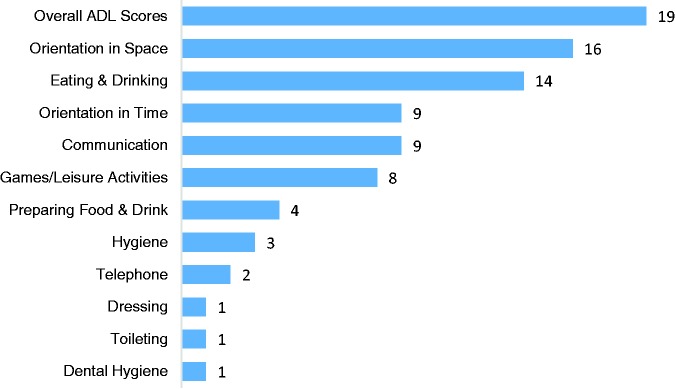
Graph displaying frequency of studies identified by the literature search by BADLS item.

Overall, the majority of the studies identified by the search were conducted in the USA (N = 48.6%), followed by Europe (N = 27.8%; N = 9.7% UK based), Canada (N = 16.7%), Australia (N = 5.6%) and Japan (N = 1.4%). Within this research 34.7% included people with moderate–severe cognitive impairment and 15.3% included people with mild–moderate cognitive impairment. Fifty per cent of the studies did not specify cognitive status or included a mixture of people with dementia who had mild-severe cognitive impairment. Eighty-five per cent of the studies identified were conducted in residential settings compared to 15% within private home environments. Overall, from the studies reviewed, specific activities from the BADLS which appeared to be most supported by environmental strategies were orientation in space and eating/drinking whereas aspects of self-care such as dressing, toileting and brushing teeth appeared to be less supported (see Figure 2). The majority of the research identified looked at the impact of environmental strategies on overall ADL performance without specifying which activities, in particular, may be benefiting from the strategy.

### Overall ADL functioning

This category encompasses studies which consider the impact of the physical environment on overall performance across all everyday activities (Table 1).

**Table 1.** Overall ADL functioning.

Author, country	Theme: Environmental strategy	Participants, setting	Research method	Main findings
Fleming et al. (2016), Australia	**Quality of environment:** Environmental auditing tool assessed safety, small size, visual access, helpful cues, clutter, familiarity, stimulating hallways, links to communal areas, private/public spaces	Two hundred and seventy-five people with mild–moderate–severe dementia (global deterioration scale) Thirty-five residential homes	Cross-sectional study, quantitative measure of ADLs (Barthel index)	Linear regression with quality of environment scores and Barthel index scores as the dependent variable revealing a significant correlation (R^2 ^= .017, P = .009)
Garre-Olmo et al. (2012), Spain	**Quality of environment:** Measured temperature, noise and lighting (morning/night)	One hundred and sixty people with severe dementia, random sample of 20 per home Eight residential homes	Cross-sectional study, quantitative measure of ADLs (Barthel index)	Linear regression with noise and Barthal index score showed significant correlation (R^2 ^= .059, P = .012), not significant for temperature (R^2 ^= .000, P = .893) and light (R^2 ^= .002, P = .651)
Milke et al. (2009), Canada	**Quality of environment:** No significant differences between environments based on therapeutic environment assessment but subtle differences in exit control, maintenance, safety, staff interaction, homeliness	One hundred and eighty four people with mild–severe dementia (MMSE score) Five dementia units, modelled on a group home	Observation (behaviour mapping) of activities over two days (14x per day)	Up to 50% differences across units in ADL activity Less engagement in ADLs at one unit (p < .0001), less ambulation at another (p < .05)
Smit et al. (2012), Netherlands	**Size:** ’Group living home characteristics’ scale measuring homelike/smaller versus larger living factors	One thousand three hundred and twenty-seven people with dementia, 12 randomly selected from each facility One hundred and thirty-six residential homes	Quantitative measure of involvement in 20 activities over three days, care staff also reported if preferred activities	More group living characteristics associated with higher involvement in activities (p < 0.001) and higher involvement in preferred activities (p < 0.001)
Verbeek et al. (2010), Netherlands	**Size:** Compared large (>20 residents) versus small scale (<8 residents) living facilities	Five hundred and eighty six people with dementia in large setting, 183 people with dementia in small setting Twenty-eight small-scale houses versus 21 large psychogeriatric wards	Functional status measured using ADL hierarchy subscale from a resident assessment instrument	Small-scale related to higher functional status (P < 0.00)
te Boekhorst et al. (2009), Netherlands	**Size:** Compared large/small scale living facilities (max. six residents in small scale, prepare own meals)	Sixty-seven group living, 97 traditional nursing home people with dementia Nineteen group living homes versus seven traditional nursing homes	Interview for the deterioration of daily living activities in dementia (IDDD) at baseline (by informal carer) and 6 min later (by staff)	Slower deterioration over six months in group living (B = −4.37, p < 0.01) and more socially engaged (B = .79, p < 0.05)
Reimer et al. (2004), Canada	**Size:** Assisted living (AL) had less residents (10) per bungalow, more staff, biodiversity (pets/plants), more homelike, functioning kitchen, small garden compared to traditional home	One hundred and eighty five people with moderate–severe dementia (global deterioration scale) Three groups: AL, two control (waiting to move to AL + staying at traditional home) Twenty-four traditional homes, four AL	Assessed quarterly for one year. Quantitative measure of ADLs (brief cognitive rating scale and functional assessment staging)	Functioning/self-care differences for AL versus traditional home (p = .012) Slower decline for those in assisted living (p < 0.016) Slowest decline for those due to move (p < 0.001)
Warren et al. (2001), Canada	**Size:** Compared residential care centre (RCC) (modelled on woodside place) versus special care unit (SCU) within nursing home	RCC 36, SCU 44 people with moderate–severe dementia Two units	Functional assessment and independence quantitative measure, observation of engagement in activities at six, 12, 18 months	Rates of decline not significantly affected by setting (p = 0.076) Observation showed residents more active in RCC (80%) versus SCU (66%)
Marquardt et al. (2011), USA	**Architectural layout**: Spatial layout (space syntax) with floor plans of homes: ‘convexity’, number of broken up rooms and ‘intelligibility’, connectivity of most used space	Eighty-two people with mild–moderate–severe dementia (MMSE score) Private home	Quantitative measures, IADLs and psychogeriatric dependency rating scale	Higher convexity associated with lower basic ADL performance (p = .02) Intelligibility not associated with ADLs Living with caregiver strongest correlate of instrumental ADLs (p = .001)
Morgan-Brown et al. (2013), Ireland	**Homelike atmosphere:** Renovated unit includes open plan design, functioning kitchen supported by carer, communal sofas (homelike)	Seventeen (pre), 18 (post) people with severe dementia (unmatched) Two residential homes renovated	Snapshot observations every 5 min for 16–28 h, six months and one year after changes	More likely to undertake an activity (p < .001) and interact with the environment independently (p < .001) post-renovation for both settings
Schwarz, Chaudhury, and Tofle (2004), Columbia	**Homelike atmosphere:** Renovations included cluster of resident rooms around living/dining areas, decentralised dining areas, private bedrooms (homelike)	Thirty people with dementia (unspecified stage/type) Long-term care facility	Focus groups with staff and observation (behaviour mapping) of activities, pre-, post and three months following renovation	Residents choosing activities, more communication/less disruption during dining, staff interacting more with residents post-renovation
Cioffi et al. (2007), Australia	**Homelike atmosphere:** New unit with private bedroom/en suite, personalised bedroom, garden access, larger windows with view and multisensory room	Seven relatives, 12 staff experiencing old/new unit Twenty-one bed dementia unit	Qualitative focus groups, three months following relocation to new unit	Themes with new unit: Better eating behaviour, spending more time outside, more sociable, design impacting residents functioning and quality of life
Wilkes et al. (2005), Australia	**Homelike atmosphere:** See Cioffi et al. (2007)	Sixteen people with moderate–severe dementia (severe MMSE) Setting, see Cioffi et al. (2007)	Quantitative measure (functional dementia scale), three and six months following relocation	No significant differences pre move (M = 61.72), three (M = 62.47) and six months (M = 61.91)
Gitlin et al. (2001), USA	**Tailored individual adaptations**: 5–90 min home visits, including environmental simplification and recommended adaptive equipment (as well as education/social strategies)	One hundred and seventy-one people with mild–moderate dementia (unclear how assessed) Private home environment	Randomised trials, 3 min after intervention and baseline, functional independence measure and IADL quantitative measures	Less decline in IADL dependence in experimental versus control (p = .03) Less ADL dependence in post-test for experimental but not significant
Gitlin, Winter et al. (2003), USA	**Tailored individual adaptations**: Expanded on Gitlin et al. (2001) study to include providing specialist equipment and low-cost environmental strategies	One hundred and eighty eight people with moderate–severe dementia (MMSE) Private home environment	Six months after intervention and baseline, functional independence measure and IADL quantitative measures	No significant differences for functioning in ADL (.927) or IADL (.685) Caregivers reported receiving fewer days of help with ADLs (.026).
Gitlin et al. (2005), USA	**Tailored individual adaptations**: Follows on 6 months after Gitlin, Winter et al. (2003) study. Over next 6 months ‘maintenance’ stage where caregivers received a home visit and three telephone sessions	One hundred and twenty seven people with moderate–severe dementia Private home environment	Twelve months after intervention, range of quantitative measures	Loss of intervention effect for days receiving help Return to baseline for measures of ADL performance
Gitlin et al. (2010), USA	**Tailored individual adaptations**: Expands on previous intervention, biobehavioural with multiple components (including environmental component suggested by OT)	Two hundred and nine people in various stages of dementia Private home environment	Four months and nine months quantitative measures following intervention as above	Four months significant improvement in functional dependence for IADLs (p = .007) in experimental No significant improvement in ADLs at 4 min Nine months loss of effects, no significant findings
Graff et al. (2006), Netherlands	**Tailored individual adaptations**: OT style intervention with 10 visits to patient/family member, first four including possibility for modifying home environment	One hundred and thirty-two people with mild–moderate dementia (brief cognitive rating scale), randomised to control or treatment condition Private home environment	Baseline, six weeks and 12 weeks follow up assessed with assessment of motor and process skills and processing section of IDDD (observation)	Six weeks: significant differences in process and performance of tasks (p < 0.0001) Twelve weeks: differences maintained (p < 0.0001)
Dooley and Hinojosa (2004), USA	**Tailored individual adaptations**: Solutions including labelling draws, adaptive devices, e.g. pill reminder box, individualised suggestions based on performance in tasks using assessment of functioning	N = 40 University outpatients with mild–moderate Alzheimer’s (MMSE 10+) Private home environment	One month after intervention and baseline, affect and activity limitation-Alzheimer’s disease assessment + physical self-maintenance scale	A significant MANCOVA main effect was obtained for caregiver burden, positive affect, activity frequency and self-care status for treatment group, F(4, 31) = 7.34, p < .001

ADL: Activities of daily living; IADLs; instrumental ADLs; MMSE: Mini Mental Status Exam.

#### Quality of the environment

Two studies were interested in looking at associations between the quality of the environment and quality of life for people with dementia. However, both included a measure of ADL performance for residents using the Barthel index and therefore calculations using the original data are reported here (Fleming, Goodenough, Low, Chenoweth, & Brodaty, 2016; Garre-Olmo et al., 2012). Linear regression modelling showed a positive correlation between environmental quality (e.g. familiarity, engaging environments, range of private/communal spaces) and Barthel index scores (Fleming et al., 2016). Garre-Olmo et al. (2012) focused on ambient features of the environment (temperature, noise, lighting) across a smaller selection of eight residential facilities and results suggested ‘noise’ was significantly associated with the ability to perform basic ADLs. Another study by Milke et al. (2009) compared five very similar facilities, modelled on a ‘household’ setting and found subtle environmental differences, (e.g. exit control, safety, homeliness), predicted up to 50% difference in engagement in everyday activities based on observations.

#### Size

A large study by Smit, de Lange, Willemse and Pot (2012) compared 136 facilities and found smaller settings with more group living/homelike characteristics were associated with significantly higher involvement in activities among residents with dementia and perhaps more importantly, involvement in preferred activities based on staff feedback. Another large study (Verbeek et al., 2010) also found living in a smaller facility was associated with higher functioning in ADLs. Verbeek and colleagues discussed this relationship in terms of people with higher functional status being selected as residents for smaller assisted living facilities, whereas Smit and colleagues discussed the reverse (the environment was causing differences) thereby demonstrating issues with determining cause-and-effect relationships here. Some studies compared small versus large facilities and functioning in ADLs longitudinally. Reimer, Slaughter, Donaldson, Currie and Eliasziw (2004) and te Boekhorst, Depla, de Lange, Pot and Eefsting (2009) found a significantly slower rate of deterioration in everyday activity performance over six months and one year for people living in smaller settings compared to larger settings. Warren et al. (2001) failed to find this effect over 18 months; however, their study was restricted to comparing two settings whereas the former two studies were comparatively larger (comparing 26–28 facilities).

#### Architectural layout

Marquardt et al. (2011) considered the spatial layout within private home environments (N = 83) in relation to ADL ability among people with dementia. They found negative associations between ‘convexity’ or more open-plan living and BADL performance (but not IADL performance) based on quantitative measures, suggesting enclosed rooms with clear functions are more supportive. ‘Intelligibility’ or connectivity of frequently used space to the rest of the house was not found to be associated with ADL functioning.

#### Homelike atmosphere

Research comparing the effects of building renovations appeared to be aimed at creating a homelike atmosphere, although they differed in the types of changes they made in order to do this. For example, Morgan-Brown, Newton and Ormerod (2013) observed differences in activity engagement pre/post changes to two dementia units which included open-plan living, a functioning kitchen and communal sofas. They found more engagement in activities and independence in activities (p < .001) post-renovation, for both settings. Schwarz, Chaudhury and Tofle (2004) employed a similar design to examine the impact of renovations such as a decentralised dining space, clustered living rooms and private bedrooms, observing that residents tended to have more choice around which activities to participate in, alternating their time between central and local living spaces. Two studies evaluated the impact of one renovated unit whereby key changes included private bedrooms, personalised rooms, garden access and larger windows. Qualitative focus groups demonstrated changes in ADL performance such as ‘better eating behaviour’ and ‘more time outside’ (Cioffi, Fleming, Wilkes, Sinfield, & Le Miere, 2007) whereas these changes were not picked up in the quantitative assessment as no significant differences in ADL performance were noted at three months or six months post-renovation (Wilkes et al., 2005).

#### Tailored individual adaptations

Gitlin, Corcoran, Winter, Boyce and Hauck (2001), Gitlin, Hauck, Dennis and Winter (2005) and Gitlin, Winter et al. (2010) have published a series of randomised controlled trials using home-based environmental interventions. The environmental component involved factors such as removing or labelling objects, the use of colour contrast, clutter management and purposeful placement of objects, although it was not specified exactly which changes were implemented. They found limited effects on ADL performance in their early study with people with moderate–severe cognitive impairment. For example, at six months they found family members reported providing significantly less help with ADL activities compared to a control group (p = .026, Gitlin, Winter et al., 2003), however this returned to baseline at 12 months post-intervention (Gitlin et al., 2005). In a later study with people with mild–moderate cognitive impairment and with an extended version of the intervention (including more time with the occupational therapist and a biological component), they found significantly higher independence in IADLs (p = .007) compared to a control group though this was lost 12 months after the intervention (Gitlin et al., 2010). It is worth noting this intervention involved an educational and training aspect for carers and therefore the exact impact of environmental adaptations on outcome measures is unknown. A similar study was conducted by Graff et al. (2006) using a more sensitive measure of ADL ability and found significant effects for the treatment group at six weeks (p < .0001) and 12 weeks (p < .0001) post-intervention; however, again this was a multi-component intervention and exact environmental changes were not specified. Dooley and Hinojosa (2004) conducted a narrower study which involved suggesting environmental solutions based upon observed performance in various everyday tasks for 40 people with dementia living at home. They found a significant group effect for this intervention on activity frequency and self-care ability.

### Multiple everyday activities

This next category includes a cluster of studies identified with specific environmental strategies for multiple everyday activities (>2) (Table 2).

**Table 2.** Multiple everyday activities.

Author, country	Theme: Environmental strategy	Sample/setting	Research method	Main findings
Chard et al. (2009), Canada	**Tailored individual adaptations**: Environmental solutions included: Labelling drawers and closer doors, visible workstations, providing ADL equipment, removing distractions, cueing and simplifying processes	Five people with moderate–severe stage dementia Assisted living facility	Observed performing two daily living tasks of their choice and evaluated using assessment of motor and process skills, post-observation after two weeks	All showed significant improvements in process ability measures/2 for motor abilities (>0.05)
Cash (2004), UK	**Assistive technologies:** Medication carousal (n = 7), locator device (n = 12), memo minder (n = 6), telephone (n = 1)	Twenty-eight people with mild dementia (16 living alone) Private homes	Product testing and subsequent qualitative feedback, semi-structured interviews, each chose a product and tested for free	Locator device was most popular, particularly when the carer was supporting use, telephone useful, Medication carousal ‘I’d be lost without it’
Evans et al. (2011), UK	**Assistive technologies:** Enabling smart technology using pre-recorded prompts/messages (from family), cooker-minder system to support living independently	One case study, female, mild dementia (MMSE, 21) Smart technology in new sheltered housing	Case study, 12 months, smart-technology installed to examine if helped live independently, qualitative feedback from family carers (quarterly) and quality of life measure	Some effectiveness, e.g. improved QOL rating in final six months, found automatic lighting useful, felt anxious about calls, returned to own home, family have installed similar devices ‘this is helping her to live independently’ (staff)
Arntzen et al. (2014), Norway	**Assistive technologies:** Simplified remote for TV and mobile phone, stove timer, night and day calendar, medicine dispenser with alarm, item locator, digital calendar, verbal reminder connected to timer for coffee machine, electronic door lock	Twelve people with mild–moderate dementia and family carers, younger sample <65 years Private home	Qualitative longitudinal, interviews and observation exploring use of assistive technologies at home, every three months for 12 months	Found supported everyday life and activities if addressed practical challenges and was user friendly The simplified mobile phone and TV remote control were considered more beneficial than the digital calendar

ADL: Activities of daily living; MMSE: Mini Mental Status Exam; QOL: quality of life.

#### Tailored individual adaptations

Chard, Liu and Mulholland (2009) conducted a study similar to Dooley and Hinojosa (2004) (Table 1) whereby they observed five people with dementia performing an everyday task (e.g. setting the table, folding laundry) and suggested environmental solutions based on their performance. Strategies such as removing clutter, simplifying the environment and using labels were found to be associated with improved performance in a subsequent observation session (p < 0.05).

#### Assistive technologies

Assistive technology research appears to be in its infancy, with studies identified using exploratory methods (e.g. product testing and qualitative feedback) and small numbers of participants (N < 28) to understand the value of technological devices for people with dementia. A locator device, medication carousel (Cash, 2004), automatic lighting (Evans, Carey-Smith, & Orpwood, 2011), simple mobile phone and TV remote control (Arntez, Holthe, & Jentoft, 2014) have been reported as particularly useful for supporting everyday activities. On the other hand, systems which automatically telephone family members for help (Cash, 2004) and a digital calendar (Arntez et al., 2014) were identified as less useful and confusing to use.

### Mealtimes

Many of the studies identified were around supporting mealtimes (preparation and consumption) (Table 3).

**Table 3.** Mealtimes.

Study, country	Environmental intervention	Sample/setting	Research design	Main findings
Josephsson et al. (1995), Sweden	**Tailored individual adaptations**: ADL selected by individual (prepare drink/set table) supported by environmental solution, e.g. removing clutter, using labels, table with model process	Four people with dementia at psychogeriatric day care unit within hospital	Baseline, post-intervention video-recording assessment using assessment of motor/process skills and frequency of support noted	Three out of four of the participants demonstrated significant gains in the ability to perform the daily activities related to meal preparation
Starkhammar and Nygård (2008), Sweden	**Assistive technology:** Device attached to stove, automatically activated when turned on, switches stove off automatically, used as heat detector, alarm system	Nine people with dementia or memory impairment and five family carers Private home	Qualitative interviews following trials with stove timer device, observed in use (50 min–1 h 50 min)	Some irritated/frustrated, e.g. system shut off before finished, enabled cooking for some who’d given up, some problems with learning how to use it
Koss and Gilmore (1998), USA	**Lighting and contrast:** Increased light intensity and visual contrast for evening meal	Thirteen people with dementia High functioning dementia unit	Baseline, intervention and post-intervention measures of amount eaten and agitated behaviours over 21-day periods	Food intake highest in intervention condition Agitation decreased in high light/visual contrast
Dunne et al. (2004), USA	**Lighting and contrast:** Baseline white plates, white cups and stainless steel flatware Experimental high contrast red plates, red cups and red flatware	Nine men with advanced dementia, (MMSE M = 2.9) Care unit at ENRM Veteran Affairs Medical Centre	Food/liquid intake, ABA design. One year later with ‘blue’ high contrast condition	Significant increase for food and liquid in high contrast intervention (P < 0.001) Follow up showed significant for ‘blue’ suggesting high contrast has effect
Brush et al. (2002), USA	**Lighting and contrast:** Enhanced lighting in dining room and table setting contrast	Eleven nursing home. Fourteen assisted living facility people with dementia (MMSE score unknown) Two live in facilities	Three-day calorie count (all three meals) and changes in resident behaviour (meal assistance screening tool) and communication outcome measure pre/post changes	Calorie count: NH, +1000 calorie average increase (p < .16); AL, (p < .01) increase **Communication:** Significant increase at NH (p < .05), AL not significant (p < .115) **Behaviour:** No significant differences apart from for distractibility (p < .05)
McDaniel et al. (2001), USA	**Quality of the environment:** Extended care (EC) dining room: lower noise, higher lighting versus refurbished Alzheimer’s Unit (AU): higher noise, lower lighting, relaxing music, no television	Sixteen people with dementia One residential facility	Five-day nutritional analysis for breakfast and lunch in the two different dining environments	Mean total intake of calories and protein was higher in AU but not sig. Fluids at breakfast higher in AU over five days (p < 0.02). Significantly higher intake on day 3 (p < .5) and day 4 (p < .2)
Slaughter and Morgan (2012), Canada	**Quality of the environment:** Measured quality of dining environment: orientation, safety, privacy, stimulation, support, opportunities for control, familiar objects, facilitation of social contact	One hundred and twenty people with middle-stage dementia (assessed by global deterioration scale) Fifteen nursing homes	Observed residents abilities to walk to the dining room and to feed themselves	Environmental features that supported functional eating (e.g. finger foods) (p = .01), personal control (p = .033) and better regulation of stimulation (p = .027) reduced hazard of eating disability
Reed et al. (2005), USA	**Quality of the environment:** Compared dining environments in residential care, assisted living (less institutional facilities) and nursing home environments	Four hundred and seven people with mild–moderate–severe dementia Forty-five assisted living facilities, US	Structured mealtime observation – observing up to five residents during single meal, looked at amount of food/fluid consumed during single meal and alertness, utensil use, etc.	Lower food and fluid intake in nursing home environments (p < 0.05) RC/AL less likely to receive treatment for eating difficulty and less physical difficulties
Perivolaris et al. (2006), Canada	**Quality of the environment:** Renovated dining area to more homelike including fireplace, bright design, smaller room, aromas of food, menu board, removed tray service style, music, staff education	Eleven people with moderate–severe dementia (MMSE) Two memory support units in long-term care facility	Baseline, six weeks after environmental renovation and further measurement at 12 weeks with staff education. Measured calorific intake, focus groups with staff, satisfaction measure with residents	Significantly more food consumed after environmental intervention (p = 0.05) and further effects with staff training (p = 0.060) Staff feedback suggested residents more relaxed, sociable in new environment
Edwards and Beck (2013), USA	**Environmental ambiance:** Introduced a large aquarium into the dining area, each with light background and eight large fish	Seventy people mostly with severe dementia (MMSE, M = 5.57) Three specialist dementia units	Body weight and food/fluid intake were weighed at baseline for two weeks, two weeks when aquarium first introduced and then once a week for six weeks with aquarium	Significant increase in food intake with aquarium (P < 0.000), increasing trend for following six weeks, significant increase in resident body weight from start to end of study (P < 0.000)
Thomas and Smith (2009), USA	**Environmental ambiance:** Music during dining based on music preferences indicated by family members	Twelve people with middle stage dementia (global deterioration scale) Fourteen bed Alz unit	A–B–A design, for eight weeks, observed for 24 meals, visual monitoring by dietician for food intake, calorie intake measured	Twenty per cent more calories consumed when familiar music was played compared to no music. Anecdotal evidence of enjoyment of music: socially engaged, stayed in dining area longer
Desai et al. (2007), Canada	**Choice:** Traditional institutional setting with food delivered on tray versus newer, homelike environment with cafeteria style waitress service	Twenty-three (traditional facility), 26 (new facility), diagnosis of probable Alzheimer’s Academic Nursing Home	Twenty-one day energy and macronutrient intakes measured and behaviour measured using London psychogeriatric rating scale	Higher 24 h P < 0.001 and dinner P < 0.001 energy intakes in new facility due to greater carbohydrate intake More energy, carbohydrate and protein intake for residents with low BMI (p < 0.05) at new facility compared with higher No significant changes in behaviour
Altus et al. (2002), USA	**Choice:** Pre-prepared plates versus family style help yourself communal serving dishes, and further condition with nurse training to praise and prompt	Five people with moderate–severe dementia (MMSE) Dementia care unit	Observation/ratings by nurse measuring participation/communication and weight following intervention	Participation increase from 10 to 24% with family style. Communication increase from 5.5 to 10.6%. Further increase with family + training of 65% (participation) and 17.9% (communication). 3/5 gained weight
Melin and Götestam (1981), Sweden	**Choice:** Meal placed on table and patients able to serve themselves. Compared with providing meals on tray in chairs for patients in corridor	Twenty-one, mixed sample (19 dementia, two schizophrenia) Psychogeriatric ward	**Communication:** Observer recorded whether or not a given patient made contact with anyone else. **Eating behaviour:** Use of utensils, glass and napkins, each observed for 15 s, four times	Significant increase in communication in experimental group (p < 0.01). Improvement in eating behaviour (p < 0.01) in experimental
Namazi and Johnson (1992b), USA	**Choice:** Placing food and snacks in accessible area on kitchen surface with domestic style versus glass door refrigerators	Twenty-two people diagnosed with probable AD; 13 in early/mid, three severe, seven unknown (clinical dementia rating score) Dementia facility	Observation of opening fridge, taking snacks, requesting snacks, requesting assistance	Visible access to fridge didn’t affect independent snacking. But poor methodological design (e.g. red tape added to door handle in domestic fridge condition to facilitate opening)

ADL: Activities of daily living; MMSE: Mini Mental Status Exam.

#### Tailored individual adaptations

Josephsson, Bäckman, Borell, Nygård and Bernspång (1995) used observation of meal preparation tasks to form the basis of environmental changes implemented for individuals. Removing clutter, using labels and providing a table with a model example were found to be associated with improved ability to prepare drinks and set the table in subsequent observations for three (N = 4) people with dementia.

#### Assistive technologies

One qualitative study looked at the use of an assistive device attached to the stove to support meal preparation for nine people living at home with dementia (Starkhammar & Nygård, 2008). Consistent with above reports of assistive technologies, the device enabled people to begin or carry on cooking. However, it was also reported to be irritating and confusing to learn how to use this technology.

#### Lighting and contrast

Koss and Gilmore (1998) found increased light intensity and visual contrast related to increased food intake for 13 people with dementia. This was replicated by Dunne et al. (2004) who focused solely on effects of visual contrast of dining ware with nine residents and found significant increases in food (>25%) and fluid (>84%) consumption for high visual compared with low visual contrast (p < .001) regardless of colour (i.e. blue or red). Brush, Meehan and Calkins (2002) compared intake in a nursing home (N = 11) and assisted living setting (N = 14) and found a significant increase in food intake for higher light levels and table contrast conditions in the assisted living setting, whereas results did not reach significance for the nursing home. This suggests other factors may impact on the effects lighting and contrast can have overall. For example, as discussed earlier, people with less severe cognitive impairment are more likely to live in assisted living facilities (Verbeek et al., 2010) which may have affected outcomes for this study.

#### Quality of the environment

Two cross-sectional studies were identified which compared eating environments across different care settings finding that ‘quality’ of the environment, particularly less institutional (more homelike features) related to increased food intake for residents with dementia (Reed, Zimmerman, Sloane, Williams, & Boustani, 2005; Slaughter & Morgan, 2012). Another study by Perivolaris, Leclerc, Wilkinson and Buchanan (2006) relates to quality and creating a more homelike dining atmosphere. They renovated the dining area in two facilities to include a fireplace, bright design, aromas of food, a menu board and more food choice. They found significantly more food was consumed for 11 people with dementia following the environmental changes (p = 0.05), with further positive effects when staff were given nutrition training. A similar study by McDaniel, Hunt, Hackes and Pope (2001) evaluated the effects of renovating a residential facility and found no significant increases in calorie intake. However, whilst some changes may be supportive such as ‘no television’ and ‘relaxing music’ during meals, the refurbished setting also included higher noise and lower lighting levels which may have counteracted effects.

#### Environmental ambiance

One study looked at the effects of introducing an aquarium with eight large fish into the dining settings of three residential facilities for 70 people with dementia (Edwards & Beck, 2013). They found there was a significant increase in food intake with the aquarium at two weeks (p < .000) and this trend continued for the following six weeks. Another piece of research was concerned with creating a pleasant acoustic environment around resident’s music preferences (Thomas & Smith, 2009). They reported descriptive results whereby playing the most preferred music during dining, compared to no music, led to a 20% increase in the amount of food consumed for 12 people with dementia.

#### Choice

Another theme emerged for mealtimes around providing choice at mealtimes. Three studies looked at the effects of serving help yourself meals versus a more institutional approach of pre-prepared plates within care settings. All found significant effects for the help yourself layout: residents with dementia consumed more (Altus, Engelman, & Mathews, 2002; Desai, Winter, Young, & Greenwood, 2007), exhibiting more positive mealtime behaviours (e.g. appropriate use of utensils and napkins) (Melin & Götestam, 1981) and communicated more at the table with fellow residents (Altus et al., 2002; Melin & Götestam, 1981). A further observation study fits this theme whereby they looked at placing snacks in accessible refrigerators around a dementia facility (Namazi & Johnson, 1992b), comparing a glass door fridge where snacks were visible to a standard fridge where snacks were not visible. They found visibility of snacks did not affect independent snacking although lack of rigor within the research design, such as adding red tape to a fridge door handle during the study as it was proving difficult to open, may affect the validity of these findings.

### Hygiene and self-care

A smaller number of articles were identified for this section including hygiene in terms of handwashing (N = 3), dental care (N = 1), dressing (N = 1) and toileting (N = 1) (Table 4). No studies were identified by the literature search concerning bathing or showering.

**Table 4.** Hygiene and self-care.

Study, country	Theme, environmental strategy	Sample/setting	Research design	Main findings
Connell et al. (2002), USA	**Tailored individual adaptations:** Care plans to support oral care, e.g. pictures, magnified mirrors, coloured cups, consistent layout	Five people with dementia MMSE scores 13–24 Significant co-morbid illnesses Veterans Nursing Home	Care plans implemented. After five days observation by research nurse, oral hygiene assessed by dentist	Observation: 4/5 become more independent in key ‘in the mouth’ oral care tasks Hygiene ratings by dentist improved 47%
Labelle and Mihailidis (2006), Canada	**Assistive technology:** Audio-verbal versus audio–video ‘automated prompting system’ to support handwashing steps	Four people with moderate dementia, four severe (MMSE) Academic nursing home	Videotaped 9 am–2 pm in washroom measuring number of handwashing steps without help and number of interactions with caregiver, counterbalanced 60 trials	No significant difference for steps completed. Significantly less interaction with caregiver for video condition (4.4) compared to audio (3.3) (p < 0.001)
Mihailidis et al. (2008), Canada	**Assistive technology:** Wall mounted computerised device, using artificial intelligence to guide through handwashing using prompts	Six people with moderate–severe dementia Long-term care facility	Alternated trials, one per day (40 trials overall), observed independence and communication	Completed 11% more handwashing steps independently, 60% fewer interactions with caregiver, Not affected by MMSE score
Boger et al. (2013), Canada	**Familiar cues:** Five different water faucet designs which varied in familiarity	Twenty-seven people with mild–severe cognitive impairment (MMSE) Long-term care facility	Fifty trial, randomly presented with the different faucet types and asked to wash hands, observed and participants provided feedback	Familiarity with taps correlated with better overall usability and lower levels of assistance from caregiver Dual lever design achieved best overall usability
Namazi and Johnson (1992a), USA	**Simplifying the environment:** Closet simplification modification, sequential arrangement of clothing	Eight people with moderate–severe dementia (clinical dementia rating, 2 or 3) Specialist dementia unit	Staff observers completed dressing questionnaire indicating independence, verbal prompts, physical prompts	Closet modification increased level of independence by 19% Verbal prompts and other assistance decreased

MMSE: Mini Mental Status Exam.

#### Tailored individual adaptations

A study by Connell, McConnell and Francis (2002) examined the impact of tailored environmental solutions to support oral care for five individuals with dementia. Strategies included using pictures as models, providing coloured cups and magnified mirrors and a consistent layout. They observed that four out of five of the residents became more independent in this task and there was a 47% improvement in dentist hygiene ratings with the environmental intervention.

#### Assistive technology

Two studies evaluated the effects of a wall-mounted prompting system to support handwashing steps by videoing trials to examine the number of steps completed by individuals with dementia (Labelle & Mihailidis, 2006; Mihailidis, Boger, Craig, & Hoey, 2008). The first of these studies compared audio and visual-audio prompting systems and found the video system was most effective in supporting people with dementia (n = 8). The later study developed the audio-visual prompting device and found people with dementia (n = 6) were able to complete 11% more handwashing steps individually with 60% fewer interactions with professional carers. Cognitive status based on Mini Mental Status Exam (MMSE) scores was not found to affect the ability to interact with the device in this study.

#### Familiar cues

Boger, Craig and Mihailidis (2013) examined the effects of five different tap designs which varied in familiarity. They observed familiarity correlated with ease of use with the dual-lever design found to be most accommodating in supporting independent handwashing. They also found familiarity of tap design was more important than cognitive status (MMSE) in predicting the ability to complete handwashing steps.

#### Simplifying the environment

Namazi and Johnson (1992a) found sequentially arranging clothes in a separate section of the wardrobe for eight people with moderate–severe dementia increased independent dressing by 19% according to staff observations. This was the only study identified around dressing and whilst it offers a simple demonstration of the effectiveness of wardrobe modification, it could benefit from replication with a larger sample.

#### Orientation to space

Although an ADL is its own right (Table 6), environmental strategies to increase orientation to space can also be used to support other activities, in this case toileting. Namazi and Johnson (1991) (Table 6) found that arrows and a sign with the word ‘toilet’ to cue people with dementia towards the bathroom were effective in supporting 44 people with dementia to enter and use the toilet independently. Picture cues were found to be less effective overall based upon these observations.

### Orientation to time

This section includes five studies using environmental strategies to cue people with dementia to the time of day (Table 5).

**Table 5.** Orientation to time.

Study, country	Theme, environmental strategy	Sample/setting	Research design	Main findings
Bailey and Haight (1994), USA	**Familiar cues:** Providing visual cue to bath day (calendar with duck picture), based on Piaget’s child development theory	Fifteen people with mid-stage dementia (3–8 on MSQ) Nursing home	Pre/post-test, recognition based on staff rating of whether recognised duck compared to other pictures and if indicated bath day	Significant increase in recognition scores for experimental group (p < .005)
Tanaka and Hoshiyama (2014), Japan	**Familiar cues:** Bright yellow tablecloth with flowers in vase on table to signify lunchtime (compared with grey tablecloth without decor), further condition with music + décor	Twenty people with dementia who had difficulty identifying mealtimes (3–18 MMSE) Elderly care facility	Repeated measures design, asked at beginning, end and few hours after ‘which meal is this?’	Significant increase in recognition of mealtime in visual condition (p < 0.001) Further significant increase in visual+auditory condition (p < 0.01) Recognition not maintained a few hours after meal
Nolan and Mathews (2004), USA	**Familiar cues:** Large clock and a sign with large lettering that identified mealtimes/time in dining area	Thirty-five people with dementia diagnosis, unspecified stage Dementia facility	Direct observation sessions before meals over five months, ABAB design. Two observers. Noted questions/comments	Less disorientation (indicated by less questioning) in intervention phase compared to no intervention before breakfast (p = .028) and dinner (p = .087) but not lunch (p = .747)
Topo et al. (2007), Norway, Lithuania, Ireland, UK and Finland	**Assistive technology:** Night and day calendar to orient to time of day	Fifty people with mild–moderate dementia (MMSE > 12) and their family caregivers Private homes	Five qualitative interviews over 12-month period after initial introduction to device	Most respondents used calendar and found useful if motivated to use it Ease of use could be improved especially for those with visual impairments
Kerkhof et al. (2015), Netherlands	**Assistive technology:** Touchscreen digital planning boards in bedrooms/living room. Daily schedule with photos of residents, overview of activities and housekeeping, visual highlights to show time	Six people with dementia, five family carers, six staff Small-scale group home	Qualitative interviews following implementation of assistive technology device over three months	Majority used planning board, advantages included confidence, peace of mind, convenience but problems with ease of use Some felt staffs job to orientate to time

MMSE: Mini Mental Status Exam.

**Table 6.** Orientation to space.

Study, country	Theme, environmental strategy	Sample/setting	Research design	Main findings
Netten (1989), UK	**Architectural layout:** Standardised measures of social, personal and architectural features administered to staff at different residential settings	One hundred and four people with moderate–severe dementia. Six group homes and seven communal homes	Staff rated wayfinding to four significant locations	**Communal homes:** Physical disability, medication, number of zones/exits, simple decision points accounted for wayfinding (p < .03) Group homes: orientation, mental ability, physical disability, light, elaborate decision point and length of routes accounted for wayfinding (p < .06).
Elmståhl et al. (1997), Sweden	**Architectural layout:** Compared units for general design, space, lighting, noise, communication area, floor plan using screening scale	One hundred and five people with dementia (MMSE 11.3–15.7) Eighteen group living units	Observed patients before, six months and one year rated using disorientation measure	Corridorlike design associated with higher disorientation/confusion overall. L-shaped corridor associated with less disorientation
Marquardt and Schmieg (2009), Germany	**Architectural layout:** Existing architectural layout of different facilities	Four hundred and fifty people with mild–severe dementia (global deterioration scale) Thirty nursing homes	Nurses rating residents’ abilities to employ wayfinding skills in five tasks based on observation	Increased number of residents affected orientation abilities but not for mild stage (p < .05) Straight circulation over changes in direction improved orientation (p < .05). One kitchen/dining area improved wayfinding (p < .05)
Namazi and Johnson (1991), USA	**Familiar cues:** Cueing to public toilet, including (1) sign ‘restroom’, sign ‘toilet’, (2) picture of toilet/tank, (3) arrows and word ‘toilet on floor’	Forty-four people with probable dementia Alzheimer’s type, early to advanced stages Two specially dementia units	Observation using checklist with three questions (look at sign, enter toilet and use toilet), any help recorded	**Descriptive statistics:** Arrows most effective for observing, entering and using toilet. Sign with word ‘toilet’ second effective. Graphic least effective Yellow background and blue writing more effective for signs
Nolan et al. (2001), USA	**Familiar cues:** Portrait-type photograph of each participant from early childhood plus large print sign with residents name on bedroom door	Three people with severe dementia (MMSE M = 5.7) Forty bed locked special care unit in residential home	Direct observation of residents’ ability to locate their room	Room finding abilities increased from 34 to 85% for all three residents. Stabilised at 100% accuracy for all residents within a few days of trial beginning
Namazi et al. (1991), USA	**Familiar cues:** Significant and familiar objects of personal memorabilia versus non-familiar objects (chosen by staff) displayed outside bedroom	Ten people with mild–severe dementia (clinical dementia rating scale) Corinne Dolan Alzheimer’s Centre	Observations of ability to find room in 3 min when asked by observer	For people with severe dementia, neither cue helped. Those in mid stages showed mixed results across cue types. Ability to locate bedroom for those mildly impaired was benefitted equally by both sets of objects on display
Gibson et al. (2004), Canada	**Familiar cues:** Environmental renovation including texture/colour/structure at entrance to each room being individualised	Nineteen veterans with moderate dementia (MMSE) Secure dementia care facility within chronic care hospital	Measured ability to find own room and intrusion into others’ rooms monitored. Pre/post, interviews	Eight-four per cent could find room first time, three couldn’t after three attempts (no p values available). Reported using structure/colour of entrance to help
Caffò et al. (2014), Italy	**Familiar cues:** Compared (1) assistive technology with lights and remote controlled sound, (2) familiar objects and right/left location instructions along two familiar routes	Four people with moderate–severe dementia (MMSE) Residential care facility	Wayfinding measured by number of sections travelled correctly and cues needed	Assistive technology significantly improved orientation over the familiar objects condition (p < .01). Familiar objects rated significantly less environmentally disruptive (p < .001)
Chafetz (1990), USA	**Distracting cues:** Grid made of black tape on white floor near exit doors	Thirty people with moderate–severe dementia (global deterioration scale scores/MMSE scores) Thirty bed dementia care unit	Frequency of door openings per 24 h measured by staff. ABA repeated measures	No effect of intervention. Found significant differences in door contacting over time (p < .001) with more contact in grid condition
Hewawasam (1996), UK	**Distracting cues:** Black insulation tapes in two different grid configurations (vertical/horizontal) on floor in front of exit doors	Ten people with various dementia diagnosis (MMSE) NHS trust hospital ward	**Observation:** Crossings versus non-crossings of the grid, repeated measures design	Horizontal grid significantly effective in reducing contact with exit door (p < .01), vertical grid less so (p < .05)
Hussain and Brown (1987), USA	**Distracting cues:** Masking tape in different grid patterns in front of exit doors, laid by authors	Eight males with dementia, one moderate dementia, seven severe (MMSE) Mental hospital ward	Observation by researchers, whether crossed grid. Repeated measures design	Eight-strip grid reduced crossing significantly from baseline (p < .01). Trend towards reduced crossing for three, four, six strips of tape (from 98 to 45%)
Dickinson et al. (1995), USA	**Distracting cues:** Horizontal mini blind and cloth barriers to cover up the panic bar/doorknob of exit doors on a corridor	Seven people with dementia	Observation of frequency of exit attempts and behavioural notes	Blind reduced exit behaviours but not significant (p < .07). Cloth barrier significantly reduced exiting (p < .001)
Mayer and Darby (1991), UK	**Distracting cues:** Full length mirror versus reversed mirror versus no mirror 1 foot from exit door	Nine people with severe dementia (MMSE < 12) Psychogeriatric ward	Observational behavioural measure of mirror/exit door contact	Less contact with door for mirror compared to no mirror (p < .02). Less contact with door in reversed mirror condition but not significant (p < .062). Higher number of approaches in mirror condition
Kincaid and Peacock (2003), USA	**Distracting cues:** Wall mural painted over exit door to disguise the doorway. Sea scene	Twelve people with dementia Special care residential unit in nursing home	Observational measure of reduced exiting behaviours at the doorway. Pre/post renovation	Significant decrease in door testing overall (p = .024) and in two particular door testing behaviours: calmly testing doors (p = .024) and employing a team effort to exit (p = .033)

MMSE: Mini Mental Status Exam.

#### Familiar cues

Bailey and Haight (1994) found pairing bath day with a pictorial cue of a duck resulted in a significant increase in recognition (p < .005) of bath time by people with dementia. Another more recent study (Tanaka & Hoshiyama, 2014) looked at enhancing visual cues in the dining area to support mealtimes. They found introducing a bright tablecloth and flowers to signify lunchtime resulted in a significant increase in recognition before and after the meal. Pairing this with music resulted in a further increase in recognition. These findings may help to explain mealtime research which suggests renovated, homelike environments relate to increased food intake; perhaps these new environments provide stronger cues to signify it is dinner time. Another study by Nolan and Mathews (2004) used a large clock and sign to signify mealtimes for 35 residents with dementia. They found significantly less questioning about time of day to staff before breakfast and dinner (not lunchtimes) with this cue.

#### Assistive technology

The only multi-country study identified for this review features here involving 50 people living at home with dementia across Norway, Lithuania, Ireland, UK and Finland (Topo et al., 2007). They conducted repeated qualitative interviews with patients and family members over 12 months of using a night and day calendar. As with other assistive devices there were some issues reported around ease of use but most found it useful overall, particularly when motivated to use it. Another study by Kerkhof, Rabiee and Willems (2015) examined the use of a digital planning board mounted to the walls in the living room and residents’ bedrooms in a group living facility in the Netherlands. The daily schedule, including mealtimes and housekeeping activities, was presented on the boards under photographs of each resident. When it was time for an activity this sounded in the living room and the activity lit up on the board. Similar to Topo et al. (2007), positive qualitative responses included confidence, peace of mind and convenience for residents; however, others had problems with use and some felt staff should tell them the daily schedule.

### Orientation to space

A large number of studies were identified around using the physical environment to support wayfinding, all within residential facilities (Table 6).

#### Architectural layout

Early research by Netten (1989) compared the layout of six group homes and seven communal homes and the wayfinding abilities of 104 residents with moderate–severe dementia, as rated by staff. They found for communal homes, simple two-way decision pathways and a smaller number of exits supported independent wayfinding whereas shorter length of routes and a clear decision point was supportive in smaller group homes. Later research by Elmståhl, Annerstedt and Åhlund (1997) found L-shaped corridors were useful for wayfinding when comparing 18 residential facilities. A more recent larger investigation by Marquardt and Schmieg (2009) compared 30 nursing homes and wayfinding skills for 450 residents with mild–severe cognitive impairment. They found straight direct routes supported orientation more than routes that required a change in direction.

#### Familiar cues

The study mentioned earlier by Namazi and Johnson (1991) is relevant here, whereby they observed the ability of people with dementia to find their way to the toilet using various cues. They found wayfinding skills were better with signs using the word ‘toilet’ and arrows, rather than familiar pictures (e.g. of a toilet). Two further studies looked at the effects of cues when residents were asked to find their way to various locations. One issue with this, in comparison to naturalistic observation, is that residents may have been less motivated to get to locations. Nolan, Mathews and Harrison (2001) found bedroom wayfinding ability increased from one-third correct trials to 100% correct within a few days when pictures of the residents as children and a sign with their name was placed on the bedroom door for three people with severe cognitive impairment. An earlier study by Namazi, Rosner and Rechlin (1991) failed to find an effect of providing familiar objects (memorabilia) outside people’s bedrooms, although trials for this study were not repeated. Two studies have explored the use of multi-sensory cues to facilitate wayfinding ability. Gibson, MacLean, Borrie and Geiger (2004) provided differential cues outside the bedrooms of 19 people with dementia, varying colour, texture, structure and location. Respondents reported using colour and structure, in particular to help them find their way, and the researchers found those who were better able to find their rooms reported using two or more environmental cues. A study by Caffò et al. (2014) compared the effects of providing familiar cues along routes versus assistive technology with lights and remote controlled sounds to cue wayfinding. They observed that four residents with dementia were significantly better finding their way using the assistive technology cues over familiar objects, highlighting the importance of developing and using new technological devices.

#### Distracting cues

A set of six early studies emerged from the literature search which involved disorientating people with dementia by providing distracting cues away from the exit door of residential facilities. These included environmental strategies such as distracting grid patterns of black tape (Chafetz, 1990; Hewawasam, 1996; Hussain & Brown, 1987) or covering up the exit door with a blind (Dickinson, McLain-Kark, & Marshall-Baker, 1995), mirrors (Mayer & Darby, 1991) or a wall mural (Kincaid & Peacock, 2003). Whilst all but one (Chafetz, 1990) found significant effects of these barriers on exit-seeking behaviour, this raises an important issue: who is this for? Whilst distracting people with dementia may be orientating them into spaces staff would prefer them to be in (with safety in mind), this may be disorientating the person with dementia if their aim is to go outside, which could cause distress and a loss of freedom.

### Leisure activities

This category includes studies which use environmental strategies to support leisure for people with dementia (Table 7). Most studies identified for this category did not specify if strategies related to an individual’s own interests and so includes leisure activities as opposed to individual hobbies.

**Table 7.** Leisure activities.

Study, country	Theme, environmental intervention	Sample/setting	Research design	Main findings
Mather et al. (1997), Canada	**Gardens:** Newly built walled, locked garden attached to facility with patio, flowerbeds, high walls and figure of eight walking path	Ten people with severe dementia Residential care home	Types and levels of disruptive and outdoor behaviours measured over summer/winter	In summer spent 54% of time sitting outside in garden, 19% of undirected walking in garden, 7% of active contact with people in garden
Murphy et al. (2010), USA	**Gardens:** Open wander garden introduced to facility with direct access from unit	Thirty-four male veterans with dementia Locked ward dementia unit	Longitudinal observational study with baseline at 12 months before garden installed, monthly measures for 12 months following introduction	Average number of visits highest when garden first introduced, dipped in winter, rose slightly in Spring but didn’t reach levels at start
Edwards et al. (2013), Australia	**Gardens:** Sensory therapeutic garden with atrium/sun room. Including memory boxes, mural, viewing platform and water feature	Ten people with dementia Specialist dementia care unit within residential care home	Interviews with staff and relatives about patient experience of using the garden. Garden usage measured by staff (behavioural observation), baseline/three month follow up	Observation: 100% of residents used the garden instead of the TV room during leisure periods Significant improvement in quality of life (p < .0001) with increased garden usage
Hernandez (2007), USA	**Gardens:** Therapeutic gardens attached to two special care units	Forty-five people with dementia Two specially designed dementia units, assisted living facilities	Observation and qualitative interviews about experience of garden	Themes relating to use of garden: actively planting in garden, goes outside and seems happy, sunbathing, relaxing and visiting garden
Detweiler et al. (2008), USA	**Gardens:** Temperate walled wander garden added to facility which is viewable to all from dining room and accessible for all mobilities. Activities organised when weather permitting	Thirty-four all male, level dementia not reported	Measured QOL, mood, behaviours and collected qualitative feedback, pre- and post-wander garden (baseline, and monthly for 12 months post garden being added)	More days in the garden predicted lower agitation scores (p < .05) Staff (72%) and family (94%) spent 15+min daily in garden with dementia residents. Ninety-six per cent of staff agreed residents enjoyed being in garden
Yao and Algase (2006), USA	**Environmental ambiance:** Measured ‘environmental ambiance’ rating how engaging and soothing environment is	Forty-seven people with dementia, +65 years, <23 MMSE score Thirty nursing homes and 17 assisted living facilities	Environmental ambiance rated by RA on site, videotaped resident movement during 12–20 min periods over two days	High ambient scores (particularly engaging environment) associated with lower frequency of walking episodes and longer sitting.
Cohen-Mansfied and Werner (1998), USA	**Environmental ambiance:** Corridor areas refurbished to be aesthetically pleasing (1) nature (including murals of forest, birdsong), (2) home (including large portrait-style photographs, music playing). Two benches in each corridor	Twenty-seven people with moderate–severe dementia (brief cognitive rating scale) identified as ‘wandering’ several times per day Two corridors within a not-for-profit suburban nursing home	Observation and electronic devices used to measure participants’ whereabouts, pacing/exit seeking/trespassing behaviours, other agitated behaviours and mood	Significantly more time spent in corridors when enhanced. More time spent sitting in corridors with simulated environments than in plain corridors. Significant increases in pleasure when in the nature corridor (p < .05)
Namazi and Johnson (1992c), USA	**Simplifying the environment:** Removable barriers of three different heights during art activity. Tallest excluded corridor from view, lower allowed some visual distractions	Twelve people with mild–moderate dementia, clinical dementia rating scale (1–3) Corinne Dolan Alzheimer’s centre	Observational data collected on duration, type and frequency of distraction behaviours (resistance to auditory and visual stimuli) during art project. Four trials with different barriers	Barriers of both heights decreased distraction by two-thirds. In those with mid and high MMSE scores distractions significantly reduced with both barrier heights (p < .0001)

MMSE: Mini Mental Status Exam; QOL: Quality Of Life.

#### Gardens

A study by Mather, Nemecek and Oliver (1997) examined behaviour when access to the garden was available in the summer compared to when it was locked in winter. They observed that in summer, 54% of time was spent sitting in the garden and only 7% was spent actively conversing with others in this space. The study by Murphy, Miyazaki, Detweiler and Kim (2010) was more robust and measured visits to a new garden area over 12 months for 34 residents with dementia. They found the garden was most used when first introduced whereas use decreased significantly over time suggesting ‘novelty’ of the garden area as a leisure space may wear off. They also found for residents in wheelchairs spending time in the garden was associated with increased agitation, highlighting the importance of taking into account individual’s health limitations. Another study by Edwards, McDonnell and Merl (2013) observed residents use of a new addition garden to a dementia facility. They found all residents used the garden space over a TV room during leisure time; however, measures were only taken at baseline and three months therefore it is unknown if this is a ‘novelty’ effect. Hernandez (2007) conducted qualitative interviews with 45 residents with dementia following the introduction of a garden at two different facilities. This space enabled activities such as planting in the garden, sunbathing and relaxing. Another study using qualitative interviews found garden space was useful for staff and family members to spend time with residents (Detweiler, Murphy, Myers, & Kim, 2008).

#### Environmental ambiance

Yao and Algase (2006) conducted a naturalistic comparison of 30 nursing homes and 17 assisted living facilities and used video recording to analyse the movement of residents within these settings. They found engaging and soothing spaces related to more sitting time and less walking. Similarly, Cohen-Mansfield and Werner (1998) found residents spent more time sitting in corridor areas after they were refurbished, with a nature setting (including pictures of forests and sounds of birdsong) rated as significantly more ‘pleasing’ than the plain corridor setting.

#### Simplifying the environment

An early study by Namazi and Johnson (1992c) looked at the effects of reducing environmental stimulation during an art activity on concentration during the task, by putting up barriers between individuals. They found higher barriers reduced distractions significantly for 12 individuals with dementia. Again, this raises the issue of whether this environmental strategy is for the resident with dementia or staff. Completing the recreational activity may be important to the teacher facilitating this activity whereas perhaps the person with dementia would prefer to be distracted by their neighbour and communicate with others around the room.

### Communication

This final category includes studies which have been mentioned in previous sections. For example, creating homelike meal settings was found to enhance communication between staff and residents with dementia (see Altus et al., 2002; Brush et al., 2002; Melin & Götestam, 1981; Nolan et al., 2001; Perivolaris et al., 2006; Thomas & Smith, 2009, Table 2). The study by Mather et al. (1997) also observed 7% active communication among staff and residents with dementia when a garden space was provided. On the other hand, some environmental solutions may be ‘disabling’ communication (e.g. Mihailidis et al., 2008; Nolan & Mathew, 2004) as they support people with dementia to independently complete everyday tasks without the need to interact with staff.

Two studies were identified where environmental strategies to enhance communication were the primary focus (Table 8). A recent study by Ferdous and Moore (2014) relates to the theme architectural layout whereby they used space syntax measures (as used by Marquardt (2011)) to explore the impact of layout on communication. Behavioural observations across three dementia facilities showed private areas with better proximity were related to more in-depth conversations among residents, whereas more visible, open spaces were related to low-level ‘small talk’. The researchers concluded that a mixture of private and public spaces is required to support different types of communication.

**Table 8.** Communication.

Study, country	Theme, environmental intervention	Sample/setting	Research design	Main Findings
Ferdous and Moore (2014), USA	**Architectural layout:** Space syntax measurement (analysing spatial configuration) looking at visibility/accessibility of spaces by configuration	Participant details not specified, 150 behavioural observations Three long-term care facilities	Behavioural observations using dementia care mapping (total 150 behavioural observations) of activities every 5 min for 6 h	Low-level social interactions in locations with better proximity/visibility (p < 0.01) but high-level social interactions in low visibility/ proximity (p < 0.01)
Kovach et al. (1997), USA	**Homelike environment:** Old: larger, nurse station located centrally, large dining and activity spaces, institutional finishes New: smaller, own dining, activity area and activity-based kitchen, personal furniture	Fourteen people with dementia Traditional nursing facility/dementia care unit	One month before and two months after move, behavioural observations, frequency counts of staff/resident beh in diff settings	Increased interaction (p < .005) and active participation (<.041) on two units. More social on dementia unit.

The final study (Kovach, Weisman, Chaudhury, & Calkins, 1997) relates to the strategy of creating a homelike environment whereby the researchers examined the impact of renovating two different facilities on interaction among residents. They found the renovations related to an increase in interaction (p < .005) for both centres demonstrating the importance of homelike atmospheres for fostering social relations.

## Discussion

Overall, this review demonstrates there is a role for the physical environment in supporting bodily performance in everyday activities among people with dementia. A diverse range of papers testing various physical environmental strategies across a range of everyday activities were identified. Given this diversity it was not possible to use existing quality appraisal measures to assess the quality of research; however, the studies were descriptively assessed for quality (similar to Caffò et al. (2013)). In reviewing and appraising this work, some important discussion points were raised around the complexity of research on the physical environment and where resources should be targeted in future to best support people with dementia.

### Researching the physical environment

The research identified aims to understand how the real-world physical environment impacts the way people with dementia externally perform everyday activities, a topic which is methodologically complex but vitally important for understanding how to design dementia-friendly supportive spaces. As with any research in real-world environments, the independent variable is co-existing with multiple other variables making it methodologically challenging to manipulate. Large-scale studies examining the effects of multiple changes in the environment or comparing multiple buildings to overall ADL performance (e.g. Fleming et al., 2016; Smit et al., 2012; Verbeek et al., 2010) provide strong evidence that the holistic environment does impact performance of daily activities for people with dementia. Longitudinal research even suggests the holistic environment may be protective in terms of slowing decline in everyday functioning for people with dementia (Reimer et al., 2004; te Boekhort et al., 2009) highlighting the importance of research into the physical environment. However, these large-scale, macro-level studies do not tell us which aspects of the holistic environment have an impact on performance, making it difficult to replicate these findings and translate them into practice settings. Furthermore, understanding how the environment is affecting activity performance is difficult; for example, ‘quality’ environments and building renovations may impact staff well-being and the way staff support residents with dementia, having an indirect effect on daily activity performance. These potentially confounding variables are difficult to control for, as summarised by Fleming et al. (2008), the psycho-social and physical environment often go hand in hand.

In contrast, studies which measure the effects of more specific changes to the environment, e.g. colour contrast crockery versus white plates for food intake (Dunne et al., 2004) (Table 3), are advantaged in demonstrating the specific environmental elements that have an impact, however they often lack methodological rigour. For example, the study by Dunne et al. (2004) was conducted with nine people with advanced dementia and limited to one setting, making it difficult to generalise findings. However, the environment is made up of infinite components and to test each, and replicate this work, would require an extensive amount of resources, possibly explaining why many environmental suggestions are based upon work with small samples or good practice guidelines (see van Hoof et al., 2010).

From the studies identified, a range of measures were used to determine effects of physical environmental strategies on activity performance making it difficult to appraise the quality of this research overall, for example using critical appraisal tools. Quantitative research provides significant data for a number of environmental strategies supporting activity performance (e.g. Marquardt & Schmieg 2009; Tanaka & Hoshiyama, 2014). Some quantitative studies used existing ADL scales to measure overall everyday activity performance (e.g. Gitlin et al., 2001; Wilkes, Fleming, Wilkes, Cioffi, & Le Miere, 2005); however, from this data it is unclear which everyday activities in particular may be benefitting from the environmental strategy. Coupled with the above issue of not knowing which environmental change has an impact, this poses a significant challenge to the applicability of such findings. Qualitative research demonstrates people’s opinions of how environmental strategies benefitted their everyday performance, providing important information, e.g. that some assistive technologies are difficult to learn to use (e.g. Kerkhof et al., 2015; Topo et al., 2007). However, people may not be able to accurately report the effects of the environment on overall daily functioning, as exemplified by Wohlwill’s (1973) report title ‘The Environment is Not in the Head!’. Observation methods may provide a more objective measure of the effects of the environmental strategy, however do not tell us how people with dementia feel about the environmental component and are open to observer bias. Ten of the studies identified by the search used a combination of measures (e.g. Brush et al., 2002; Edwards et al., 2013; Schwarz et al., 2004) which may be most effective, albeit time consuming, for this type of research.

A further complication when researching the physical environment for people with dementia concerns ‘novelty’ effects of new environments. Behaviour (of people with dementia and/or carers) may change in association with an environmental change, but individuals may become habituated to these new environments, with functioning in everyday activities returning to baseline in time (e.g. Murphy et al., 2010). This is further complicated by the fact dementia is a progressive disease; therefore, it is unknown how people with dementia may habituate or respond to environmental strategies in time. Some of the research identified for review was conducted longitudinally for over 12 months (e.g. Murphy et al., 2010; Warren et al., 2001); however, the majority was cross-sectional and longer term follow-up research may be necessary. Cross-sectional studies are further complicated by the fact there may be key between-group differences which may explain differences in ADL performance (e.g. Smit et al., 2012; Verbeek et al., 2010).

### Applicability of the Powell-Lawton model

Theoretically, many of the studies identified drew on Lawton & Nahemow (1973) ‘Press-Competence Model’ to justify the use of physical environmental strategies. However, this model was originally designed in relation to ageing as opposed to dementia. With this in mind, many of the studies focused on cognitive function (e.g. MMSE score) to measure competence. However, dementia is a multi-faceted disease which can affect multiple other cognitive domains including memory, language, perception and decision making as well as motor functions. Furthermore, different types of dementia syndromes exist and there is a lack of specification in the studies identified regarding the specific disease profile.

The linear model designed by Powell-Lawton may not be detailed enough to capture the dementia experience and in turn, understand the effects of the environmental strategies to support these. This is supported by the fact that some studies did not find MMSE scores related to the effectiveness of environmental solutions (e.g. Labelle & Mihailidis, 2006; Mihailidis et al., 2008). Perhaps to move forward with this type of research, a more complex model taking into account the full ‘neuropsychological profile’ of individuals with dementia combined with ‘environmental press’ (Lawton & Nahemow, 1973) and psycho-social factors is needed. This is supported by a recent review (Giebel et al., 2015) which highlights there is a lack of knowledge about the error patterns associated with individual everyday activities for people with dementia.

Providing tailored environmental strategies for individuals which support specific areas of difficulty may be the most effective way to facilitate ADLs, given profiles of dementia can vary considerably from person to person (e.g. Gitlin, Winter et al., 2005, 2003; Graff et al., 2006). For example, using written signs to support wayfinding may be helpful for a person with exclusive memory impairment whereas visual contrast and pictorial cues may support someone who in addition has perceptual difficulties due to dementia.

### Future directions

From the literature search, it appears activities such as mealtimes and orientation in space are particularly well researched in this field, whereas other daily activities such as self-care and dressing may be overlooked (Figure 2). This may be due to mealtimes being reported as a significant problem for people with dementia, e.g. increasing weight loss (White, Pieper, & Schmader, 1998) and/or functioning in this activity being easier to measure, e.g. food intake, over personal activities such as showering. Hobbies were one activity which appears to be overlooked by this research, as no studies were identified using the physical environment to support people with their preferred leisure activities. This raises the important issue around where resources should be targeted to help people with dementia (and carers) to live well. An article by Harmer and Orrell (2008) examined meaningful activity for people with dementia within residential settings and found activities which support psychological and social needs are important for individuals with dementia, therefore supporting hobbies with environmental strategies may be particularly useful. Personhood is important here, in terms of recognising an individual’s needs and desires around the daily activities that are most important to him/her. Future qualitative research could work more with people with dementia in being a part of developing and discussing environmental strategies.

Following on from this, studies should be clear about who the environmental strategy is for. For example, some residential studies may be more supportive for staff as opposed to people with dementia. For example, research around disorientating people with dementia away from exit doors (e.g. Chafetz, 1990; Hewawasam, 1996) may be seen as helpful for staff as opposed to people with dementia, whereby such strategies may cause distress or confusion. Often strategies designed to increase independence in activities, by definition, reduce social interactions. For example, adding a digital planning board reduces the need for residents to talk to staff about the day’s activities (Kerkhof et al., 2015). This may have a negative impact on the quality of life of people with dementia, where engaging with others in this context may be socially important. Staff are an important aspect of the holistic residential environment, therefore providing solutions which support all setting participants may be advantageous; however, studies should be clear on this focus.

Given 85% of the studies identified were conducted in residential settings where people in more severe stages of dementia are likely to live (Knapp et al., 2007), this raises issues around whether supporting independence in everyday functioning is most appropriate. Environmental strategies in residential settings may be better targeted towards supporting affect or overall well-being (e.g. Gitlin, Liebman et al., 2003; Padilla, 2011) rather than activity performance outcomes. Supporting people at home in earlier stages with environmental strategies for activity performance may be particularly useful, both in terms of maintaining their self-identity in everyday tasks and for supporting family carers in terms of reducing stress and carer burden (Graff et al., 2006). However, entering the home space for environmental research is overwhelmingly complex given these spaces are highly variable. Nevertheless, research such as by Dooley and Hinojosa (2004) and Marquardt et al. (2011) demonstrates possibilities for this type of research.

## Conclusion

To conclude, this is an important area of research which appears to support a role for the environment in assisting people with dementia to perform a range of everyday tasks. Future studies could include individuals with dementia in developing environmental strategies to further understand where to focus resources to best support ADL function and related quality of life and well-being (Andersen et al., 2004). Theoretical understanding of how people with dementia interact with the environment during activities may also help in designing specific environmental adaptations. Models would need to incorporate the multi-faceted nature of dementia which differs across individuals. Targeting neglected spaces such as the private home and activities such as hobbies and personal care may also be beneficial. Overall, this research area is rich with plenty of opportunities for growth. The wide range of studies demonstrates opportunities for developing a battery of design concepts which can support individuals with dementia in daily life.
